# Perceptions of Healthcare Providers and Caregivers Regarding Procedures for Early Detection of Developmental Delays in Infants and Toddlers in Saudi Arabia

**DOI:** 10.3390/children9111753

**Published:** 2022-11-15

**Authors:** Afnan Sohail Gmmash, Nada Osama Faquih

**Affiliations:** 1 Department of Physical Therapy, Faculty of Medical Rehabilitation Sciences, King Abdulaziz University, P.O. Box 80200, Jeddah 21589, Saudi Arabia; 2 Department of Speech-Language Pathology and Audiology, Faculty of Medical Rehabilitation Sciences, King Abdulaziz University, P.O. Box 80200, Jeddah 21589, Saudi Arabia

**Keywords:** screening, early detection, well-child visits, developmental delays, healthcare providers, infants and toddlers

## Abstract

Background: This study aimed to explore current practices adopted by frontline healthcare providers for the early detection of developmental delays in infants and toddlers in Saudi Arabia, with a specific focus on motor and speech delays and caregivers’ perceptions of early detection of developmental delays and their awareness of well-child visits. Methods: Two cross-sectional surveys were conducted and distributed. The first survey was performed among healthcare providers who worked directly with infants and toddlers in the first 3 years of life, and the second survey was performed among caregivers of infants and toddlers who received healthcare services. Participants were recruited via online groups, social media platforms, and clinics. Results: Overall, 60% of the healthcare providers played a role in identifying medical conditions that could be associated with delays and disabilities. However, they did not consistently check for normal development or developmental delays. Furthermore, the healthcare providers reported low frequencies of documenting developmental growth. The caregivers’ survey results showed that 67% were familiar with the concept of “well-child visits”, and the most frequently discussed topic by the healthcare providers was motor development. Conclusions: Well-defined, government-supported standards are needed to encourage regular well-child visits and implement efficient practices for the early detection of developmental delays.

## 1. Introduction

In their first few years of life, children rapidly develop motor, speech, and language skills. Various motor skills are learned in the first year, starting with head control to taking first steps [1,2]. Similarly, speech and language skills go through multiple stages of development during the first year, from beginning to perceive and vocalize sounds to producing the first word at 12 months of age [3]. Signs of delay in motor, speech, and language development may appear in as early as the first months of life; these delays could influence other areas of development such as social and cognitive development [4,5]. Motor, speech, and language delays can be obvious and indicate some pediatric conditions such as hypoxia or genetically predisposed conditions that can be diagnosed prenatally, perinatally, or immediately postnatally [6]. Although eligibility criteria might differ from one place to another, early identification of certain conditions such as severe cerebral palsy, severe global developmental delays, and failure to thrive independently qualifies some infants for early rehabilitation services [7]. These early intervention services may have a tremendous positive effect on infants’ later development [8,9]. However, conditions such as autism spectrum disorder, mild cerebral palsy, and developmental delays owing to rare diseases cannot be identified prenatally and may lead to further delays in infants in the first 3 years of life. These delays may be dismissed by parents and caregivers if the infants are not regularly screened by their pediatricians or primary healthcare providers [10]. It should be noted that primary health care systems vary between countries. One of the factors contributing to these variations is the difference in training programs of physicians and how they are prepared to provide primary and screening services for pediatrics [11]. While, in the United States, usually pediatricians and family medicine practitioners are responsible for well visits and developmental monitoring and screening, in Australia general practitioners play this role [12], In Europe, differences occur across countries with either the general practitioners or the primary care pediatricians providing these services [11]. Therefore, the term health care providers will be used throughout this paper to refer to frontline individuals providing health care services for infants and toddlers.

Regular screening during well-child visits is critical for detecting any delays that could result from undiagnosed medical conditions or inappropriate child-rearing practices [13]. Thus, some countries have implemented specific developmental monitoring (DM) and developmental screening (DS) guidelines to observe infants’ social, emotional, language, communication, cognitive, and physical development [14,15]. These two processes are highly recommended for the early identification of developmental delays. Regarding DM, healthcare providers monitor infants’ development by conducting routine assessment and asking caregivers relevant questions. Conversely, healthcare providers use specific validated tools in DS to identify potential delays formally [14,15]. The U.S. Preventive Services Task Force and the American Academy of Pediatrics (AAP) guidelines are some of the existing guidelines for DS and developmental surveillance of infants and young children. The AAP recommends that healthcare providers use standardized tools to assess the development of infants and toddlers at 9, 18, and 30 months of age. Additional screening for motor development is recommended at 3, 6, and 12 months [15]. The AAP also suggests that infants should be screened for speech and language development delays during well-child visits at 9, 18, and 30 months because speech and language skills are typical developmental landmarks at this stage. Formal checklists and questionnaires are available for this purpose [16]. Of note, the hearing of newborns should be checked at birth as part of the newborn hearing screening program and then at 4, 5, 6, 8, and 10 years of age during well-child visits [17]. Generally, the AAP recommends the establishment of a medical home for each child. A medical home is a form of continuous care that highlights the importance of a main care provider for children [18]. The presence of a medical home allows for regular follow-up and ensures that each child receives the required services. A great deal of efforts was recently made by the Ministry of Health (MOH) to improve the health care systems in Saudi Arabia. The MOH initiated a program for neonates to test them for metabolic diseases, and endocrine diseases, congenital heart disease, and hearing tests. In addition, the MOH provided an awareness platform in their official website to raise the public’s health literacy. For example, there is a detailed guideline for the continuous monitoring of the infants’ development on the MOH’s website under the awareness platform. Additionally, there is a baby growth manual available on the same page in the MOH’s website to educate parents on red flags and provide them with a detailed schedule for important checkups. However, this page is only available in Arabic, and the number of reads for this page is low [19].

There are strict strategies followed in Saudi Arabia regarding childhood vaccinations. There are vaccination schedules and certificate that are posted on the MOH’s website, and children are required to have vaccination records to enter schools in Saudi Arabia. These strict policies could have led to the increased rates of immunized children in the country and in the acceptability of childhood immunization by the Saudi parents [20,21,22,23]. However, to our knowledge, there is a lack of a specific policy that guides motor and speech development in the early years of life. An absence of a government-supported regular screening program could lead to the dismissal of undetected motor and speech delays [24]. The application of both DS and DM strategies for early detection increases the chances of infants and children receiving early interventions and special education services [16,24]. Early access to therapeutic services could be beneficial to not only infants but also caregivers and the community. Early detection and diagnosis could accelerate the attainment of goals, improve parental satisfaction, and enhance children’s motor development. Caregivers can play a proactive role in detecting developmental delays by communicating their concerns to healthcare providers to begin with appropriate interventions. There are multiple resources available online to support caregivers in monitoring their children’s development. For instance, the Centers for Disease Control and Prevention published a checklist to assist caregivers in tracking their children’s development from 2 months to 5 years [17]. Early detection is key to initiating early intervention services. Delayed provision of rehabilitation services can hinder a child’s overall development, leading to long-term consequences.

In Saudi Arabia, increasing recognition has been directed toward early identification and intervention of certain disorders. Some associations such as “Mobakker” also support early intervention services [25]. A detailed proposed guideline on early intervention programs was also published by the Ministry of Education and King Abdullah Bin Abdulaziz Public Education Development Project in Saudi Arabia [26]. However, there is no current evidence or known active initiatives suggesting that such programs were implemented. The website of the MOH of Saudi Arabia recently included a general guide about the important tests that should be made for infants and toddlers in the “Child Health” section of its educational webpage [27]. Nevertheless, it is not mentioned that a specific program is initiated and applied to support these guidelines. Therefore, further evidence is needed to design a protocol for monitoring and screening motor and speech development in infants and toddlers aged 0–3 years and measure the protocol’s efficacy. Therefore, this study conducted an in-depth pilot interview among pediatricians to obtain an overview of the current practices of performing well-visits and early detection of developmental disabilities in Saudi Arabia. We also conducted a survey to explore current practices conducted by healthcare providers and caregivers in terms of awareness of early detection procedures. We believe that our study will provide preliminary data that will aid the development and implementation of well-child visits and programs in Saudi Arabia to detect developmental delays, with a specific focus on speech and motor delays.

## 2. Materials and Methods

### 2.1. Study Design

This cross-sectional survey used convenience sampling. Only individuals residing in Saudi Arabia were included in this study. Two online surveys were created by the investigators: one for healthcare providers and the other for caregivers.

### 2.2. Recruitment Process

A link to the online survey and a barcode were distributed to healthcare providers in Saudi Arabia. An introductory message that included the survey link, target population, and information on the research team was disseminated through multiple social media platforms and WhatsApp groups. The same message was printed and distributed, along with the link and barcode that directed them to the survey, to hospitals in Jeddah city.

### 2.3. Instrumentation

#### 2.3.1. Pilot Interview

A pilot in-depth interview was conducted for two experienced pediatricians to explore the current screening and monitoring protocols to aid in the early detection of speech and motor delays in infants and toddlers in Saudi Arabia. Two surveys were developed according to the results obtained from the pilot interviews and literature review on the same topic.

#### 2.3.2. Healthcare Providers’ Survey

The survey was reviewed by two physical therapists and two speech-language pathologists for grammar, writing, clarity, order, and question type. Ethical approval for this study was obtained from the Faculty of Rehabilitation Sciences of King Abdulaziz University. An electronic survey was developed using Google Forms and pilot tested among 10 participants. The purpose of the study; operational definitions of motor delay, speech delays, infants and toddlers, and early intervention; time to complete the survey; number of sections; and consent to participate were specified on the cover page of the survey. The survey was divided into three sections. The first section consisted of 16 questions on demographic information such as sex, age, years of experience, educational qualifications, and specialty. The second section included 16 questions on early detection and intervention practices such as frequency of well-child visits until the age of 36 months, discussion of growth charts, and most common reasons for clinic visits. The final section included 4 open-ended questions related to the referral process, protocol followed when a delay was detected, and any challenges or additional comments.

#### 2.3.3. Caregivers’ Survey

The caregivers’ survey was reviewed by a physical therapist and a speech-language pathologist for grammar, writing, clarity, order, and question type. The survey was also reviewed by six parents/caregivers. The purpose of the study, duration of the survey, number of sections, and contact information of the research team were stated on the cover page.

The parents/caregivers were asked to complete the survey based on their overall experiences with their youngest child. The first section included 5 questions related to the caregiver’s/parent’s age, educational qualification, nationality, location where the services were provided, and number of children. The second section included 20 questions related to well-child visits; the definition of well-child visit was provided at the beginning of this section. The last section included a question regarding additional comments.

#### 2.3.4. Data Analysis

The data were expressed as frequencies and percentages. Moreover, descriptive analyses in the forms of cross tabulations were used to describe the participants’ tendencies to choose some responses more than others and how their chosen responses may imply how healthcare providers apply early detection practices. Pearson chi square was also used to test for significance differences between the categorical variables (*p* < 0.05). Descriptive analysis also assisted in identifying which areas need further attention in future studies in the possibility of improving early detection practices in Saudi Arabia.

## 3. Results

### 3.1. Healthcare Providers

A total of 33 participants responded to and agreed to participate in the study. Three participants were excluded because they only treated adult patients. Thus, a total of 30 participants were included in the study; most of them were male (57%). The age of the participants ranged from 25 to 30 years (37%) and 36 to 40 years (23%). Most participants (87%) were Saudi. Moreover, 83% obtained their first medical degree from Saudi Arabia; 77% obtained their highest degree from an institution in Saudi Arabia; and 100% were currently practicing in Saudi Arabia. Most participants (82%) were currently practicing in the Western region. The participants were mainly residents (46%), followed by consultants (37%) and specialists (13%). The majority were family medicine specialists (43%), followed by general pediatricians (23%). Moreover, 75% were working in the government sector. Please refer to Table 1 for full description of the demographics. 

Early detection practices from health care providers’ perspective are shown in Table 2, indicating the most common reasons for receiving infants and young children in the clinic are illness and immunization and less frequency of receiving them for well-visits. Most participants (60%) indicated that their role in detecting a motor or speech-language delay was to identify medical conditions (spina bifida, craniofacial anomalies, and clubfoot) that could be associated with delays and disabilities, but they did not detect delays per se. Regarding early detection and intervention practices, approximately 50% of the participants stated that they either “know that there are no early intervention services” or “did not know if early intervention services for children with or at risk for motor/speech delay were provided in their current practice”, especially to those in the first 3 years of life. There was variability in the participants’ knowledge regarding the age at which early intervention was provided in Saudi Arabia. The participants agreed that the two main reasons for clinic visits in the first 3 years of life were illness and immunization. Furthermore, 24% of the participants recommended four or more well-child visits for healthy infants and toddlers (0–3 years), while 69% recommended four or more well-child visits for high-risk infants and toddlers (0–3 years). A higher number of participants stated that they always “conduct DS at the age of 24 months” (23%) than those at 36 months (21%), 9 months (20%), 18 months (20%), and 30 months (13%). Only 20% of participants always “document and maintain a developmental history at each well-child visit”; 23% “identify risk factors at each well-child visit”; 30% “assess percentiles of the growth chart’s curve at each well-child visit”; 30% “explain to the caregiver the child’s growth chart at each well-child visit”; and 10% “provide educational resources related to typical development or red flags (for example, brochures, websites, videos)”.

### 3.2. Caregivers

A total of 111 caregivers agreed to participate in the survey. Two participants were excluded from the study because one had no children and one received child services only outside Saudi Arabia. Most participants (56%) were aged between 30 and 40 years, and 55% had 2–3 children. Almost all of the participants (91%) had an undergraduate degree. Approximately, 71% of the caregivers stated that the age of their youngest child is between 0 and 5 years and 17% of caregivers stated that the age of their youngest child is between 6 and 10 years.

### 3.3. Early Detection Practices

Early detection practices from caregivers’ perspectives are shown in Table 3, indicating that the caregivers’ perspectives agree with the health care providers’ perspectives, i.e., the top reasons for visits are for due to illness and immunization. Parents indicated that the top areas covered in well-visits are: checking for are height, growth and immunization respectively, while top area discussed were motor development then child’s growth. Overall, 73% of the participants reported having monthly prenatal visits, with 80% reporting that there were no complications during pregnancy. Only 30% of the participants who had prenatal complications reported that the doctors had explained to them how the complications could affect their child’s development. Most participants (67%) reported familiarity with well-child visits.

Approximately, 41% of the participants took their infants and toddlers fewer than five times for a well-child visit during the child’s first 3 years of life. Regarding the reasons for the visits, 73% visited the pediatrician when their child was sick or needed immunization (82%). Less than half of the participants visited the pediatrician for a regular checkup (31%) or assessment of growth rate (35.8%). Moreover, 46% of participants did not have a consistent healthcare provider for their child since the child’s birth. The rest of the participants who had a consistent healthcare provider reported that the main reasons for choosing them were a good reputation and family/friends’ recommendations. They reported that the topic most frequently discussed by their child’s healthcare provider was normal motor development (58%), and the least frequently discussed topic was the recommended frequency of well-child visits (15%). Only 29% of the participants reported discussing language and speech development during the visits. Almost 90% of the participants asked their child’s healthcare provider if they had concerns regarding their child’s development. Most of the caregivers stated that they seek the guidance of pediatricians (70%) or the physicians with which they are following up (20%) when they have a concern regarding their child’s development.

Cross tabulation analysis was conducted to test the relationship between some variables. Table 4 shows the values for having one specific doctor since birth by number of children, caregiver’s educational level, hearing about well visits, and referrals. Caregivers who have a higher educational level and larger number of children seemed to be more likely to have one health care provider since birth for their children. However, this relationship was not significant. The results indicate that there is a significant relationship between having one specific health care provider since birth and hearing about well visits. There is also a significant relationship between continuously having one health care provider since birth and receiving a referral to other health care providers when needed. 

## 4. Discussion

This study aimed to explore the current procedures that healthcare providers and caregivers adopt, especially during well-child visits, to ensure early detection of developmental delays in infants and toddlers in Saudi Arabia. The results of this study provide initial evidence for the need to raise awareness about implementing early detection practices more consistently in the health care system in Saudi Arabia. Further interesting findings are discussed below.

In this study, healthcare providers stated that they “usually” screened the children for developmental delays, with the highest percentage for choosing “usually” was for implementing DS using standardized testing at 18 months old (53.3%), and (46.7%) chose “usually” for implementing DS the ages of 24 and 30 months. The option “always” was less frequently selected across all ages. These findings are not in parallel with the recommendations that DS should be conducted as early as 10 months old so children can benefit from early intervention if needed [17]. Both the healthcare providers and caregivers reported that the main reasons for visits were illness and immunization. This finding is in accordance with the results outlined by Uddin et al., who reported that there was a higher frequency of problem-focused visits than well-child visits in young children [28]. However, both the caregivers and healthcare providers did not select “well-child visits” or “concerns regarding development” as the top reasons for the clinic visits. This may lead to delayed detection and, consequently, delayed intervention. According to the 2017 census conducted by the General Authority of Statistics in Saudi Arabia, 2.9% of the Saudi population had a severe disability, and motor disabilities accounted for 29.13% of the disabilities in this population [29]. These findings emphasize the need for regular screening for disabilities during well-child visits in Saudi Arabia. Based on the caregivers’ reports, healthcare providers discussed motor developmental milestones more frequently than speech-language milestones. The reason behind this difference is unclear, suggesting the need for a holistic approach to screening. Further, more than 30% of the caregivers in this study had never heard of well-child visits. In addition, the number of actual visits varied greatly depending on the children’s ages, with only 25% reporting the use of well-child visits more than seven times in the first 3 years of life. The AAP recommends 16 well-child visits from 0 to 6 years [30]. Interestingly, healthcare providers reported much lower and inconsistent recommendations regarding the number of well-child visits for healthy infants and toddlers, with the majority recommending three well-child visits during the first 3 years of life, which is not compliant with international recommendations [15]. The percentage increased for high-risk infants or toddlers. This could be alarming, as it could mean that healthcare providers have not been provided with a unified guideline on the recommended number of well-child visits in the sensitive period of development. In addition, this could lead to the dismissal of mild cases or cases not listed in the high-risk population, so the conditions may remain undetected. Moreover, half of the healthcare providers who participated in this study lacked knowledge regarding the provision of early intervention services in Saudi Arabia. This could lead to further delays in intervention, as most caregivers stated that they depend on healthcare providers when they have concerns. Lack of compliance with the recommended number of well-child visits has been noted globally. One of the contributing factors to this lack of or reduced attendance to well-child visits is the absence of health insurance [31]. However, in Saudi Arabia, healthcare is free in governmental clinics and hospitals; thus, cost should not be an issue. This finding is consistent with the result of a study conducted by Goedken and colleagues who found that insurance does not influence the frequency of visits, but the caregivers’ educational background could [32].

Despite the abundance of free educational material available on the MOH’s website, more than 75% of the caregivers/healthcare providers stated that they did not receive any educational material or information related to normal development during the visits. In addition, the majority of caregivers who participated in our study seemed to rely on pediatricians for guidance which demonstrates the critical role of health care providers in educating and directing parents to support their children’s development. Further, more than half of the caregivers stated that they did not have a consistent healthcare provider for their infant/toddler since birth. This result does not follow the recommendation that each infant should have a “medical home” and receive consistent care from the same healthcare provider or at least the same facility to maintain a complete medical record of the child [19]. In the absence of a medical home, even if a healthcare practitioner noticed a sign of delay in the infant or toddler, he/she would not be able to screen/monitor the child if the caregiver did not return for a follow-up visit. Having the same source of care for young children is critical in tracking the development and identifying any abnormalities, changes in behavior, or signs of possible risk factors [31]. Caregivers must be educated on the importance of seeking medical care from the same sources throughout their child’s developmental period. To avoid any adverse consequences from changing pediatricians, caregivers should retain the paper or electronic records of their child’s development [33].

There are noticeable advances in the use of artificial intelligence in Saudi Arabia to accomplish Saudi Arabia’s 2030′s vision [34]. There is an increasing use of mobile applications to schedule and track vaccination, and display the health and education status of the children to their guardians. This increasing use of artificial intelligence in Saudi Arabia contributed to improving vaccination rates and lowering the rates of COVID-19 [35,36]. There is also a free reminder service for vaccination under the MOH E-services that operate to remind parents about their children’s immunization schedules [37]. The same reminding system can be used to notify parents about the schedules of well visits. In addition, the same mobile application that is currently used to track health, educational and tourist services can be used to track the children’s development based on the health data obtained from the well visits. This could be a cost effective and applicable service to accelerate the detection of developmental delays and referrals to early intervention services.

### Limitations

The limited sample size and use of the convenience sampling method may limit the generalizability of the results. However, efforts were made to collect responses from a diverse group of participants by sharing links on various platforms. Future studies should be conducted with large sample sizes and in different regions of Saudi Arabia. Some factors may have affected the study’s outcomes. For instance, many of the healthcare providers who participated in this study were family medicine practitioners. Although family medicine practitioners play an important role as frontline healthcare providers for infants and toddlers, based on our data, caregivers often seek pediatricians rather than family medicine practitioners for any concerns regarding the infant’s health.

Finally, this study was conducted between 2021 and 2022 during the COVID-19 pandemic. According to Nguyen et al. (2022), the pandemic affected the number of well-child visits, with one-quarter of parents delaying or canceling their appointments [38]. Thus, these conditions may have affected the number of well-child visits in the present study. Nevertheless, the questions regarding well-child visits were not limited to the past 2 years.

## 5. Conclusions

Our preliminary evidence indicates the need for a mandatory protocol for frontline healthcare providers in Saudi Arabia. This protocol should support systematic early detection practices of DM and DS for all types of developmental delays. The healthcare providers’ and caregivers’ responses indicated that the infants and toddlers did not undergo adequate well-child visits in their first 3 years of life. Providers should encourage caregivers to increase the frequency of visits for a complete assessment of their infants’ or toddlers’ health and development. Caregivers reported seeking medical care from inconsistent sources, which may delay the identification of risk factors. Educational materials related to normal development were rarely distributed to caregivers during the visits. This could affect the caregivers’ health literacy and their ability to advocate for their children’s health.

## Figures and Tables

**Table 1:** Participants’ demographics.

Healthcare Providers	N	(%)	Caregivers	N	(%)
Gender			Age		
Male	17	57%	18–23	1	1%
Female	37	43%	24–29	10	9%
			30–35	38	35%
			36–41	23	21%
			42–47	22	2%
			>47	14	13%
Age			Number of children		
25–30	11	37%	1	17	16%
31–35	4	13%	2	34	31%
36–40	7	23%	3	27	25%
41–45	4	13%	4	12	11%
≥46	4	13%	5	6	6%
			6	10	9%
			>6	3	3%
Nationality			Nationality		
Saudi	26	87%	Saudi	99	91%
Other	4	13%	Other	10	9%
Specialty			Highest level of education		
General pediatrician	7	23%	High school	5	5%
Family medicine	13	43%	Diploma	5	5%
Pediatric neurologist	4	13%	Bachler	55	51%
General physician	1	3%	Masters	31	29%
Pediatric plastic surgeon	1	3%	PhD	12	12%
Pediatric physiatrist	1	3%			
Pediatric Geneticist	1	3%			
Neonatologist	1	3%			
Pediatric ENT	1	3%			
Region			Region		
West	23	82%	East	6	6%
Central	5	18%	West	89	82%
			South	3	3%
			North	1	1%
			Central	10	9%
Years of experience			Relationship to the child		
1–23 months	6	2%	Mother	75	67%
2 years	1	3%	Father	27	25%
3 years	4	13%	Grandmother	5	5%
4 years	2	7%	Grandfather/other	2	2%
More than 5 years	17	57%			

**Table 2:** Early Detection Practices from Health Care Providers’ Perspective.

Healthcare Providers	N	(%)
**Role in detecting a motor or speech-language delay**		
Identify medical condition but do not detect a delay	18	6%
Receive patients who are identified in the high-risk group for evaluation	10	33%
Receive patients who are at risk for speech/motor delay for evaluation	14	47%
Well baby clinic/evaluate developmental delay	4	13%
**The starting age for receiving early intervention services**		
0–6 months	5	16.7%
7–12 months	6	20%
13–23 months	10	33.3%
24–30 months	7	23.3%
31–36 months	1	3.3%
After 36 months	1	3.3%
**Most common reason(s) infants and young children visit clinic in their first three years of life**		
Illness	19	63%
Immunization	18	60%
Caregivers’ concern about the child’s development	12	40%
Well visits upon pediatrician’s recommendations	12	40%
Well visits without the pediatrician’s recommendations	4	13%
**How often do you see the infants for the following reasons?**		
**Illness**		
Always	11	37%
Usually	16	53%
Rarely/Never	3	10%
**Immunization**		
Always	15	50%
Usually	4	13%
Rarely	5	17%
Never	6	20%
**Recommended well visit for healthy infants and toddlers**		
1	6	20%
2	7	23%
3	10	33%
4	2	7%
5	3	10%
more than 5	2	7%
**Recommend well visits for infants for higher risk infants and toddlers**		
1	3	10%
2	1	3%
3	5	17%
4	4	13%
5	4	13%
more than 5	13	43%
**How often the following are performed by the health care providers you visit:**		
**Document and maintain a developmental history at each well-child visit.**		
Always	6	20%
Usually	17	57%
Rarely	7	23%
**Identify risk factors at each well-child visit**		
Always	7	23%
Usually	16	53%
Rarely	7	23%
**Assess percentiles of the growth chart’s curve at each well-child visit**		
Always	9	30%
Usually	15	50%
Rarely	5	17%
Never	2	3%
**Explain to the caregiver the child’s growth chart at each well-child visit.**		
Always	9	30%
Usually	12	40%
Rarely	6	20%
Only upon the caregiver’s request	3	10%
**Provide educational resources related to typical development or red flags (e.g., brochures, websites, videos)**		
Always	3	10%
Usually	12	40%
Rarely	9	30%
Never	3	10%
Only upon the caregiver’s request	3	10%

**Table 3:** Early Detection Practices from Caregivers’ Perspective.

Caregivers	N	(%)
**Frequency of well visit during the first three years of life**		
0	2	2%
1	13	13%
2	9	9%
3	9	9%
4	10	10%
5	5	5%
6	7	7%
7	6	6%
more than 7	25	25%
I don’t know what well visits are	15	15%
**Reasons that make you take your child to the doctor in their first three years**		
Regular check-ups	34	31%
Doctor recommended well visit	19	17%
sick visits	79	73%
Immunization	90	83%
To check your child’s growth	39	36%
Other reasons	10	9%
**Areas consistently discussed by physicians with caregivers**		
Normal motor development	63	58%
Normal speech-language development	40	37%
Importance of well visits	24	22%
How often they should see your child	16	15%
Comparing your child’s growth to the norm	60	55%
Expectations for developmental milestones	41	38%
None of the above	18	17%
**Areas that are usually covered during well visits**		
Track how your child is growing	77	71%
Take child’s weight	98	9%
Take child’s height	93	85%
Check motor development	61	56%
Check speech-language development	32	29%
Immunization	74	68%
Ask about child’s general behavior	24	22%
Talk about how to handle medical emergencies	21	19%
Talk about how to handle sudden illnesses	19	17%
Others	6	6%
None of the above	4	4%

**Table 4:** Values for Having One Specific Doctor Since Birth by Number of Children, Caregiver’s Educational Level, Hearing About Well Visits, and Referrals.

Caregivers’ Survey				
		Having One Specific Doctor Since Birth		
		No (% of Total)	Yes (% of Total)	Raw Total
Number of children	1	6%	9%	15%
	2	16%	16%	32%
	3	8%	17%	25%
	4	7%	4%	11%
	More than 4	8%	9%	17%
Column Total		45%	55%	100%
Pearson Chi square	0.38			
Education level	High school or less/Diploma	5%	5%	10%
	Bachelor degree	25%	26%	51%
	Master/PhD	16%	23%	39%
Column Total		46%	54%	100%
Pearson Chi square	0.7			
Have heard of well visits	No	19%	13%	32%
	Yes	27%	41%	68%
Column Total		46%	54%	100%
Pearson Chi square	0.042			
If needed, Referred to other health care providers for further assessment	No	16%	10%	26%
	Yes	21%	49%	70%
	Referral was given but parent did not go	3%	1%	4%
Column Total		40%	60%	100%
Pearson Chi square	0.043			

## Data Availability

The data presented in this study are available on request from the corresponding author. The data are not publicly available due to participants’ confidentiality.
